# Clinical Remission in Severe T2‐High Asthma in Real Life After Anti‐IgE, Anti‐IL‐5 and Anti‐IL5R: A Potential Role for CRP as a Biomarker

**DOI:** 10.1002/clt2.70164

**Published:** 2026-04-15

**Authors:** Jeanne Vervier, Marie Sabbe, France Louis, Catherine Moermans, Françoise Guissard, Carole Sanchez, Virginie Paulus, Monique Henket, Genevieve Philippe, Casper W. H. Beijnink, Pierre‐Olivier Bridevaux, Florence Schleich, Renaud Louis

**Affiliations:** ^1^ Department of Pneumology University of Liege Liege Belgium; ^2^ Department of Pneumology Valais Hospital Sion Switzerland; ^3^ Department of Cardiology Radboud University Medical Center Nijmegen the Netherlands

**Keywords:** biologics, biomarkers, CRP, severe asthma

## Abstract

**Background:**

Biotherapies have transformed the management of severe asthma (SA), shifting treatment goals from disease control toward achieving clinical remission. This study aimed to evaluate the effectiveness of biotherapies in inducing remission in severe asthma and to identify baseline patient characteristics that could predict remission.

**Methods:**

We conducted an observational, retrospective, monocentric study including severe T2‐high asthmatic patients who had initiated their first biological therapy (omalizumab, mepolizumab or benralizumab) between 2006 and 2023. Clinical remission at 12 months was defined by the absence of exacerbations, no chronic oral corticosteroid use, and good symptom control (Asthma Control Test score ≥ 20 and Asthma Control Questionnaire‐6 score < 1.5).

**Material:**

Data were extracted from the registry of our asthma clinic. We included 206 patients—97 treated with omalizumab, 71 with mepolizumab, and 38 with benralizumab.

**Results:**

Of the 206 patients, 62 (30%) achieved remission at 12 months. The mean age was 52 years, and 39% were male. Remission rates were 29% for omalizumab, 28% for mepolizumab, and 37% for benralizumab. Patients achieving remission had better baseline lung function (pre‐ and post‐bronchodilator FEV1% predicted and FVC % predicted, *p* = 0.01), lower CRP levels (*p* = 0.01), earlier disease onset (*p* < 0.05), and were less likely to have a history of smoking (*p* < 0.01) and denied SABA and LAMA use compared to patients who did not achieve remission. Only CRP remained significant predictor after multiple logistic regression (*p* = 0.04).

**Conclusion:**

Biological treatments with omalizumab, mepolizumab or benralizumab are capable of inducing clinical remission at 12 months in around one third of severe T2‐high asthmatics. A low baseline CRP might be predictive of achieving remission.

## 1 Introduction

Management of severe asthma (SA) has been greatly improved by the arrival of biotherapies. These are monoclonal antibodies prescribed as complement to background treatment in cases of inadequate disease control.

SA is defined by the need of high doses of inhaled corticosteroids (ICS) to maintain disease control and by disease worsening when high‐dose inhaled corticosteroid therapy is reduced. Uncontrolled asthma is associated with persistent symptoms, exacerbations requiring oral corticosteroids, admission to hospital or deterioration of lung function [1].

Clinical remission includes good symptomatic control, assessed by validated questionnaires, absence of exacerbations requiring oral corticosteroids (OCS) or hospital visits [2]. Some authors also include functional remission which generally means no significant decline in lung function while others require a normalization of FEV1 with predicted value above 80%. Nevertheless, there is a consensus that a follow up of at least one year is necessary before concluding that remission has occurred and that clinical remission means no maintenance OCS, no exacerbation and good asthma control as reflected by ACT ≥ 20 and/or ACQ6 < 1.5.

Asthma encompasses several immune‐inflammatory phenotypes which were found to be associated with a variable response to biologic treatment in RCTs. It has become classic to differentiate asthmatics in T2 high and T2 low phenotypes. Markers of T2‐high inflammation include blood eosinophils and sputum eosinophil count, nitric oxide in exhaled air (FeNO) and atopic background best approximated by total serum IgE. The choice of biotherapy is generally based on phenotype, disease severity and comorbidities.

Leveraging our large asthma clinic data base and taking advantage of a clear care path imposed by the public authorities to reimburse the biologics in Belgium [3] (Supporting Information S1: Figure S1), we tried to evaluate the proportion of patients achieving clinical remission at 12 months in our severe T2 asthmatics who were prescribed biologics focusing on anti‐IgE and anti‐IL‐5 and anti‐IL5 (R).

## 2 Methods

### 2.1 Patient Characteristics

We conducted a retrospective longitudinal study. We selected severe T2 high asthmatics recruited aged of at least 18 years from the asthma clinic of CHU Liege between September 2006 and August 2022 in whom a biologic was justified according to the criteria used in Belgium [4]. All included patients had severe asthma with functional impairment (Forced Expiratory Volume in 1 sec [FEV1] < 80%), had experienced at least two hospitalizations for severe asthma or exacerbations managed on an outpatient basis but documented within the past 12 months, or had been corticosteroid‐dependent for more than 6 months. Additionally, they had received two sessions of therapeutic education administered by specialized nurse.

A total of 206 SA were included in this monocentric study and their baseline feature are given in Table 1. The considered biologics were omalizumab, mepolizumab and benralizumab as very few patients were on reslizumab whereas dupilumab and tezepelumab were not reimbursed in Belgium at this time.

**TABLE 1:** Baseline characteristics of severe asthma patients included in the study according to the remission status at the end of the 12 months observation period.

	Whole cohort (*N* = 206)	Group NON remission (*N* = 144)	*N* for analyses	Group remission (*N* = 62)	*N* for analyses	*p* value
Demographics						
Age (y), mean +/− SD	52 ± 15	53 ± 14	144	50 ± 17	62	0.37
Gender (male, *n*, %)	81 (39%)	54 (37.5%)	144	27 (44%)	62	0.44
BMI (kg/m^2^), mean +/− SD	27 ± 5	27 ± 5	144	26 ± 5	62	0.11
Atopy (*n*, %)	98 (55%)	67 (52%)	130	31 (66%)	47	0.12
Smoking status (%)						
Never (*n*, %)	109 (53%)	67 (47%)	144	42 (68%)	62	< 0.01
Ex (*n*, %)	76 (37%)	58 (40%)	144	18 (29%)	62	0.04
Current (*n*, %)	21 (10%)	19 (13%)	144	2 (3%)	62	0.02
Age at onset (y), mean +/− SD	28 ± 20	31 ± 20	59	22 ± 21	29	0.03
Duration of asthma (y), mean +/− SD	22 ± 17	20 ± 17	59	25 ± 19	28	0.16
Level of asthma control						
OCS burst in past year (*n*), mean +/− SD	2.65 ± 2.11	2.57 ± 2.21	144	2.82 ± 1.85	62	0.67
Hospitalization (*n*), mean +/− SD	0.27 ± 0.67	0.28 ± 0.66	134	0.25 ± 0.67	61	0.76
ACT score, mean +/− SD	12 ± 5	11 ± 4	117	15 ± 5	52	< 0.01
ACQ6 score, mean +/− SD	2.79 ± 1.24	3.11 ± 1.13	117	2.08 ± 1.21	52	< 0.01
Pulmonary function						
FEV1 (%predicted pre BD)	68 ± 19	66 ± 19	141	73 ± 17	57	< 0.01
FEV1 (%predicted post BD)	73 ± 19	71 ± 20	133	79 ± 17	55	0.02
FVC (%predicted (pre BD)	80 ± 17	78 ± 17	140	85 ± 15	57	< 0.01
FVC (%predicted post BD)	83 ± 18	81 ± 18	105	89 ± 15	45	< 0.01
FEV1/FVC pre BD (%)	69 ± 12	68 ± 13	141	70 ± 11	57	0.33
BDR (% baseline)	9.18 ± 12.37	9.25 ± 13.18	134	9.01 ± 10.25	55	0.26
Inflammatory biomarkers						
Geometric mean of FeNO (ppb), mean +/− SD log^e^ scale	31 ± 0.96	28 ± 1.00	123	37 ± 0.82	54	0.05
Geometric mean sputum neutrophils (%), mean +/− SD on log^e^ scale	43.77 ± 0.37	41.38 ± 1.05	84	49.11 ± 0.69	41	0.98
Geometric mean sputum eosinophils (%), mean +/− SD on log^e^ scale	3.94 ± 1.09	3.89 ± 0.96	84	4.05 ± 0.93	41	0.86
Geometric mean blood neutrophils count (1/μL), mean +/− SD on log^e^ scale	4.70 ± 0.37	4.87 ± 0.37	132	4.34 ± 0.36	54	0.82
Geometric mean blood eosinophils count (1/μL), mean +/− SD on log^e^ scale	325.34 ± 1.10	391.61 ± 1.15	132	356.24 ± 0.97	45	0.50
Fibrinogen (g/L), mean +/− SD	3.68 ± 1.1	3.72 ± 1.09	97	3.54 ± 1.13	30	0.43
Geometric mean CRP (mg/L), mean +/− SD on log^e^ scale	2.79 ± 1.28	3.45 ± 1.27	100	1.68 ± 1.20	41	0.01
Geometric mean total IgE (kU/L), mean +/− SD on log^e^ scale	164 ± 1.33	155 ± 1.32	122	191 ± 1.37	44	0.34
Treatment						
EQ_Beclometasone (mcg/j), mean +/− SD	2000 (2000–2500)	2000 (2000–2500)	104	2000 (2000–2100)	55	0.39
ICS, (*n*, %)	204 (99%)	144 (100%)	144	60 (97%)	62	0.22
LABA, (*n*, %)	199 (97%)	141 (98%)	144	58 (94%)	62	0.20
LAMA, (*n*, %)	37 (18%)	33 (23%)	144	4 (6%)	62	< 0.01
SAMA, (*n*, %)	86 (42%)	68 (47%)	144	18 (29%)	62	0.2
SABA, (*n*, %)	146 (71%)	112 (78%)	144	34 (55%)	62	< 0.01
THEO, (*n*, %)	8 (4%)	8 (6%)	144	0	62	0.11
LTRA, (*n*, %)	102 (50%)	72 (50%)	144	30 (48%)	62	0.88
HRA, (*n*, %)	45 (22%)	27 (19%)	144	18 (29%)	62	0.14
OCS, (*n*, %)	23 (11%)	20 (14%)	144	3 (5%)	62	0.55
Omalizumab, (*n*, %)	97 (47%)	69 (48%)	144	28 (45%)	62	0.72
Mepolizumab, (*n*, %)	71 (34%)	51 (35%)	144	20 (32%)	62	0.66
Benralizumab, (*n*, %)	38 (19%)	24 (17%)	144	14 (23%)	62	0.32

*Note:* Results are expressed as mean ± SD, geometric mean ± SD for inflammatory biomarkers, or median (range) for ICS dosage. Red indicates total in the group of non remission (144) and remission (62).

Abbreviations: EQ = equivalent, FeNO = fractional exhaled nitric oxide, FEV1 = forced expiratory volume, HRA = histamine releasing activity, ICS = inhaled corticosteroids, LABA = long acting beta agonist, LAMA = long acting muscarinic antagonist, LTRA = leukotriene receptor antagonist, OCS = oral corticosteroids, SABA = short acting beta agonist, SAMA = short acting muscarinic antagonist, THEO = theophylline.

### 2.2 Study Design

The Severe Asthma Clinic registry at the CHU Liege prospectively collects routine clinical parameters from patients with severe asthma (European Respiratory Society/American Thoracic Society criteria) at baseline and at follow‐up visits, as previously described. Follow up at the Asthma Clinic generally includes a baseline visit (T0), then regular visits every 6 months. Informed consent was obtained from all participants, and the study was approved by the local ethics board.

**FIGURE 1:**
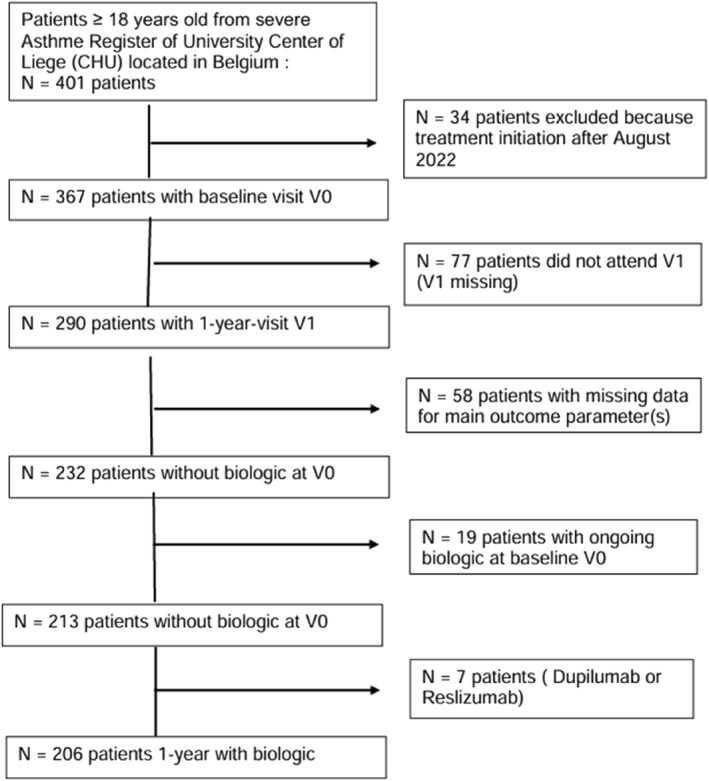
Flow chart of the patient selection.

Flowchart of patient selection is shown in Figure 1. In august 2023 we selected all patients from the registry who were aged 18 years or older in whom treatment with biologics had been initiated (*N* = 401), had received for at least 12 months (*n* = 367) and had attended the visit at one year (*n* = 290). Then, we extracted patients for whom core data were available at T0 and after 1 year (V1), including exacerbations, OCS use, asthma control test score (ACT/ACQ‐6) (*n* = 232). Patients already receiving a biologic at the time of inclusion in the registry were also excluded (*n* = 213), as were patients on dupilumab and reslizumab leaving 206 patients for the final analysis.

### 2.3 Outcome

The primary outcome of this study was to evaluate the rate of clinical remission obtained under biological treatment in severe T2 high asthmatic patients who received treatment with biologics (monoclonal antibodies directed against IgE, IL‐5 or IL‐5 receptor). Remission was defined by the absence of exacerbations, the absence of systemic corticosteroid use (chronic or intermitted) and good symptomatic control defined by an Asthma Control Test (ACT) score ≥ 20 and an Asthma Control Questionnaire (ACQ‐6) < 1.5. Exacerbation is defined as the need of systemic corticosteroids for ≥ 3 days, a doubling of the maintenance OCS dose or a visit to the emergency department/hospitalization for asthma. Patients who meet all criteria after 12 months of treatment are considered to be in clinical remission.

The secondary objective was to analyze predictive factors of clinical remission.

### 2.4 Statistical Analyses

Continuous variables were described as mean ± SD in case of a normal distribution and geometric mean in case of non‐normal distribution.

Inflammatory parameters, specifically c‐reactive protein (CRP), IgE, eosinophils, neutrophils, and FeNO were presented with geometric means with standard deviations. For sputum eosinophils a value of 0.1% was assigned when the actual value was 0.

Categorical variables were presented as number with a percentage. Categorical variables were compared using the Pearson chi‐square test whereas continuous variables were compared using the Student *T*‐test and Mann Whitney *U* test, as appropriate. To evaluate the association of CRP and FEV1 with clinical remission, a logistic regression model was fitted with CRP and FEV1 as the independent variables and clinical remission as the dependent variable.

## 3 Results

### 3.1 Patient Characteristics

The clinical characteristics of the study population at the start of the study are presented in Table 1. The mean age was 52 years with a dominance of female that represent 61% of the population. The mean body mass index (BMI) was 27 kg/m^2^ indicating overweight in the majority of patients. A majority were atopic (55%) and only 10% were current smokers while 37% had a past smoking history.

Virtually all patients were receiving ICS/LABA high dose and 11% were on maintenance OCS. On top of ICS/LABA half of the patients were receiving LTRA, 18% LAMA and 22% H1 receptor antagonists.

Patients displayed moderately altered spirometric values with mean post bronch FEV1 of 73% predicted and a mean ratio FEV1/FVC of 69%. As for T2 biomarkers, the patients had geometric mean of 31 ppb, 3.9%, 325/μL and 164 KU/L for FeNO, sputum eosinophils, blood eosinophils and IgE respectively.

The distribution of the initiated biologics was as follows: 97 patients (47%) received omalizumab while 71 (34%) and 38 (19%) patients received mepolizumab and benralizumab respectively.

The baseline characteristics of patients treated with anti‐IgE Versus anti‐IL‐5 and anti‐IL‐5 R are given in Supporting Information S1: Table S1.

### 3.2 Comparison of Baseline Characteristics in Patients Achieving Remission Versus Those Failing

Overall 62 patients achieved clinical remission after 12 months including 28 patients with omalizumab (29%), 20 patients with mepolizumab (28%) and 14 patients with benralizumab (37%) (Table 1). When a criterion for functional remission was added—defined as post‐bronchodilator FEV_1_ > 80% predicted or an increase in pre‐bronchodilator FEV_1_ > 100 mL, as defined by Pavord et al. [5] and Milger et al. [6]—remission was observed in 20 patients receiving omalizumab (21%), 16 receiving mepolizumab (22.5%), and 9 receiving benralizumab (24%). Using this four‐component definition, the overall remission rate was 22% (45 patients).

When comparing, in the whole cohort, those achieving remission versus those failing, we found that remission was associated with never smoking status (68% vs. 47% in ever smoking, *p* < 0.01) and earlier disease onset (*p* < 0.05). Greater FEV1 and FVC were found in the remission group compared to the non remission group (*p* < 0.01). As for airway and blood biomarkers, we only found that CRP levels were lower (*p* = 0.01) and FeNO levels higher (*p* = 0.05) in those achieving remission. No significant difference were noted for other T2 biomarkers. A higher proportion of patients were on LAMA and using SABA in the group failing to achieve remission (*p* < 0.01).

### 3.3 ROC Curves for FEV1, CRP and FeNO

**FIGURE 2:**
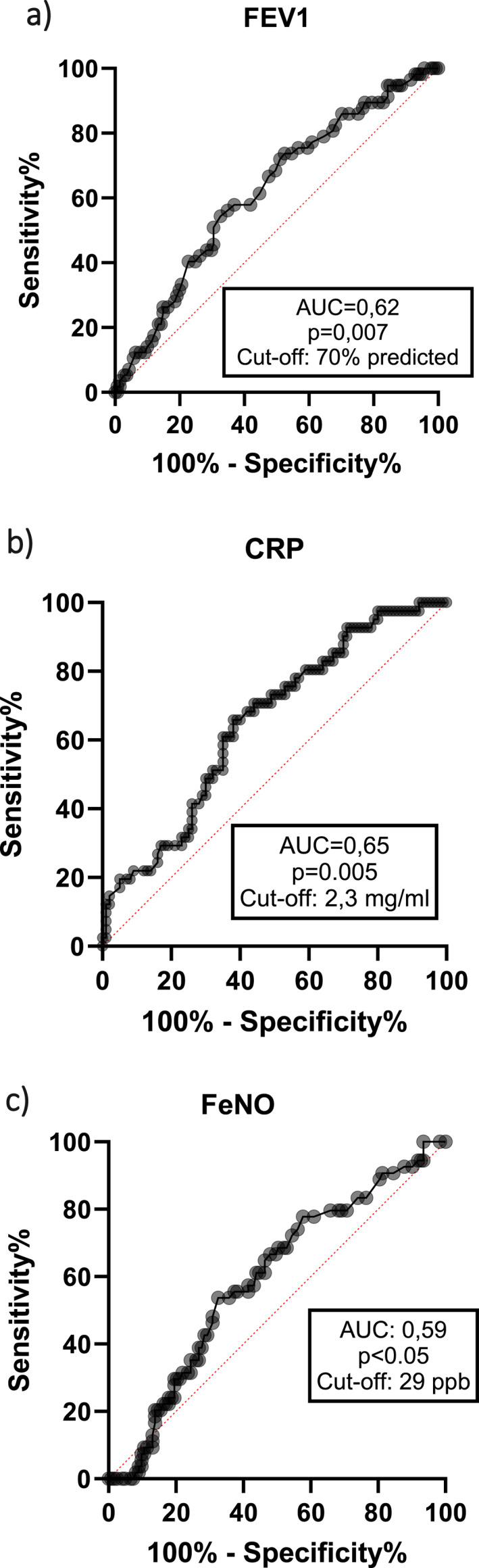
ROC curves for FEV1 (prebronch % predicted) (a), CRP (mg/L) (b), FeNO (ppb) (c).

A receiver operating curve (ROC) analysis was performed for continuous variables that showed differences between the remission and the non remission groups. The area under the curve (AUC) for FEV1 was 0.62 (*p* = 0.007) with a modest sensitivity (63%) and specificity (58%) at the Youden index of 70% predicted (Figure 2a).

Similarly, ROC curve for CRP provided an AUC of 0.65 (*p* = 0.005) with a Youden index at 2.3 mg/mL associated with a sensitivity and a specificity of 62% and 61% respectively (Figure 2b). The ROC for FeNO provided an AUC of 0.59 (*p* = 0.049) with a Youden index at 29 ppb giving a sensitivity and specificity of 67% and 52% respectively (Figure 2c).

### 3.4 Univariate and Multivariate Logistic Regression

**FIGURE 3:**
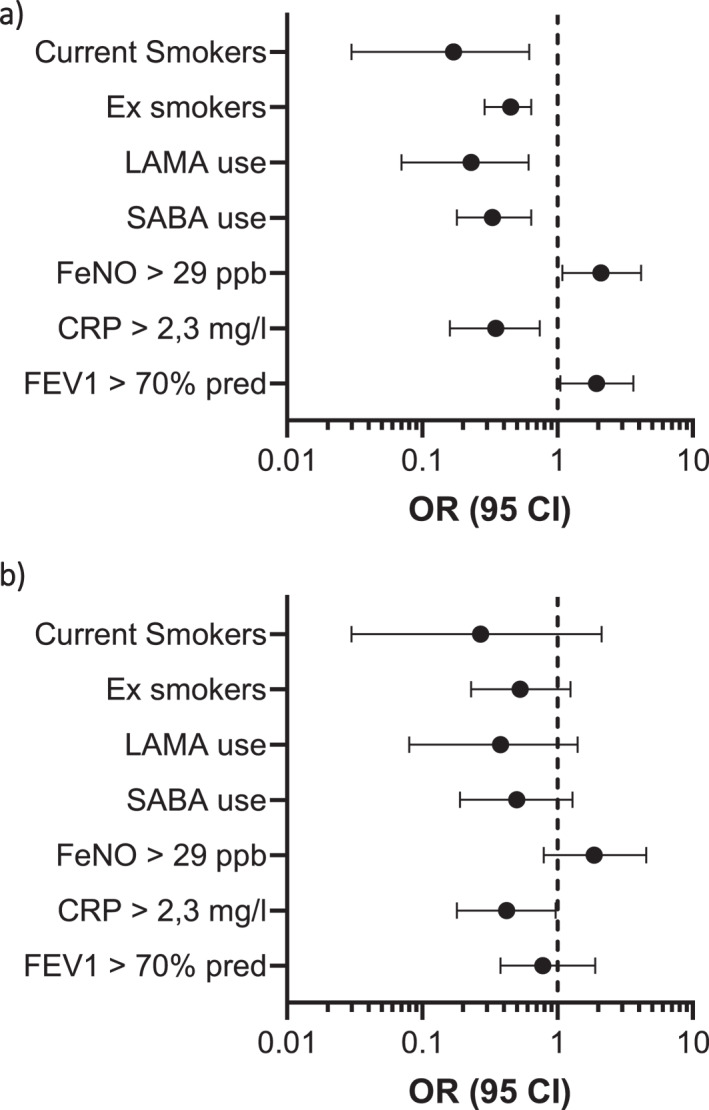
Forest plot for univariate (a) and multivariate analysis (b) OR (95 CI) Odds ratio (95% confidence interval), CRP, creactive protein; FeNO, fraction exhaled nitric oxide; FEV1, forced expiratory volume/sec; LAMA, long acting antimuscarinic agent; SABA, short acting β2 agonist.

In the univariate analysis where continuous variables were dichotomized based on the Youden index FEV1 and CRP were significant predictors together with smoking status, SABA and LAMA use whereas FeNO failed to achieve statistical significance (Figure 3a) (Table 2). In a multivariate logistic regression model including the factors found to be significant in the univariate model, only CRP remained significant predictor (OR: 0.42; 95% CI 0.18–0.97; *p* < 0.05) while FEV1 was no longer statistically significant (OR: 0.78; 95% CI: 0.38–1.90; *p* = 0.58) (Table 2) (Figure 3b).

**TABLE 2:** Factors associated with remission in the univariate and multivariate logistic regressions.

	Univariate OR (CI)	*p* value	Multivariate OR (CI) (*N* = 124)	*p* value
Pre bronch FEV1% dichotomized^a^	1.94 (1.05–3.65)	0.04	0.78 (0.38–1.90)	0.58
CRP dichotomized^a^	0.35 (0.16–0.74)	0.007	0.42 (0.18–0.97)	0.04
SABA	0.33 (0.18–0.64)	0.0008	0.50 (0.19–1.29)	0.15
LAMA	0.23 (0.07–0.61)	0.008	0.38 (0.08–1.41)	0.18
FeNO dichotomized^a^	2.1 (1.09–4.16)	0.029	1.87 (0.79–4.54)	0.16
Smoking status				
Past	0.49 (0.25–0.94)	0.03	0.54 (0.23–1.25)	0.28
Current	0.17 (0.03–0.62)	0.02	0.27 (0.04–2.12)	0.11

Abbreviations: CI = confidence intervals, FeNO = fractional exhaled nitric oxide; FEV1 = forced expiratory volume, LAMA = long acting muscarinic antagonist, OR = odds ratios, SABA = short acting beta agonist.

^a^ CRP > 2.30 mg/mL; FEV1 > 70% predicted; FeNO > 29 ppb.

Among the 46 patients with CRP > 5 mg/L at baseline, only 8 (17%) achieved remission. Using the 5 mg/L as a threshold for CRP gave a significant predictive value in an univariate model with an odds ratio (95 CI) of 0.37 (0.17–0.87) (*p* < 0.05) but not in the multivariable model with an odds ratio (95 CI of 0.45 (0.16–1.14) (*p* = 0.11).

## 4 Discussion

In this real‐life study we found that approximately one third of severe T2 high asthmatics started with anti‐IgE or anti‐IL‐5 and anti‐IL‐5 R achieved clinical remission 1 year after initiation. Greater proportion of non smoking history and of no SABA use, higher spirometric values, lower CRP but higher FENO levels at baseline were found in the remission group compared to the non remission group. In multivariate analysis low CRP levels remained significantly associated with remission.

To date, the definition of remission is still a matter of debate. In this study we opted for the pragmatic definition which is most often used, as proposed by Menzies Gow et al. [7] Therefore, we chose not to include FEV1 as a remission criterion for which it not decided yet whether it is better to choose between attaining a fixed % predicted threshold or preventing a further FEV1 decline.

The treatments considered in this real life current study were omalizumab, mepolizumab and benralizumab. Our overall remission rate of 30% is in line with the remission rate of several other real life studies using similar criteria to define remission. Based on real‐life data from Germany Milger et al. identified 37% of patients in remission after 1 year of treatment [6]. A recent systematic review and meta‐analysis, which has analyzed clinical remission rates defined by a three‐component definition including use of maintenance oral corticosteroids, exacerbations and asthma symptom burden, pooled data from randomized control trials and real life studies found an overall remission rate of 38%. [8] In the current study the remission rate achieved in the omalizumab group is slightly higher than in the Australian study by Thomas et al. Indeed they found a rate of 23% but adopted a slightly more stringent definition of symptom control (ACQ‐5 score ≤ 1) [9]. Our remission rate with mepolizumab is in line with that reported in the literature as found by Maglio et al. who reported 30% of remission using a similar definition [10]. While 37% of our patients achieved remission with benralizumab, the retrospective multicenter XALOC‐1 study found a 43% remission rate [11] and the study by Martinez et al.reported a remission rate of 44%, while taking lung function into account (FEV1 ≥ 80%) [12].

In our study, adopting the four‐component definition of remission, which includes lung function, reduced the remission rate to approximately 20%, in keeping with other studies [8, 9].

We sought to determine whether demographic, functional and inflammatory biomarkers might be predictive of achieving clinical remission. Our finding of SABA use, altered FEV1 and smoking history being associated with less chance of achieving remission is in keeping with previous studies [13]. In our study, the remission rate was not clearly predicted by T2 biomarker levels, although FeNO showed borderline results, with higher baseline values observed in the group that experienced a favorable outcome.

This aligns with the results of the Menigoz et al. who reported that patients with baseline FeNO levels ≥ 50 ppb had a greater exacerbation reduction at 12 months compared with those displaying levels < 50ppb. [14] The lack of predictive value of T2 biomarkers may appear surprising in view of our previous report [15] but is likely explainable by the fact that the patients had already been selected as being T2 high by the reimbursement criteria, so leaving less room to show convincing prediction of remission as we defined it. It might certainly have been different if we had included FEV1 improvement in our definition of remission and investigated patients with less pronounced T2 disease.

The original and intriguing finding of our study is the relationship between the CRP levels and the prediction of remission. To the best of our knowledge, this association had not been described yet. In our cohort, patients in remission had significantly lower CRP levels at baseline. CRP levels is the most solid biomarker among those investigated in the current study. The predictive value persisted after multiple logistic regression. CRP, which is synthesized by the liver, is a marker of an IL‐6‐dependent inflammatory process. Our data suggest that in cases of IL‐6‐mediated inflammation, accompanied by elevated CRP levels, biological treatments may be less effective. This mechanism could partly explain the lower response to biologics observed in patients with obesity [16], a condition which often goes along with chronic low‐grade inflammation and elevated CRP levels. [17]. However, this is not observed for another marker of systemic inflammation like fibrinogen.

Our study has some limitations. First it is a monocentric study. Second it is retrospective with the pitfalls inherent to that design such some missing values. However, the stringent care path imposed by our public health authorities for the reimbursement of biologics serves us in shaping consistent data base. The aim of our work was not to perform direct comparisons of the treatment efficacy, as baseline demographic or immuno‐inflammatory characteristics could differ between groups according to the type of biologic, making strict comparison difficult. This would require a “propensity score matching” technique to select certain patients within each group and make the baseline characteristics of the patient groups fairly comparable. Such comparison would therefore require larger and multicentric cohorts.

## 5 Conclusion

Biological treatments with omalizumab, mepolizumab or benralizumab are capable of inducing clinical remission at 12 months in around one third of patients with severe T2‐high asthma. A low CRP level at baseline might predict the occurrence of remission so that it might be become an interesting biomarker to look at when initiating a treatment with anti‐IgE of anti‐IL‐5 and anti‐IL‐5R.

## Author Contributions

**Jeanne Vervier:** conceptualization, investigation, funding acquisition, writing – original draft, methodology, validation, visualization, writing – review and editing, project administration, resources, supervision, data curation, software, formal analysis. **Marie Sabbe:** investigation, funding acquisition. **France Louis:** methodology, conceptualization. **Catherine Moermans:** conceptualization, investigation, methodology, software, data curation. **Françoise Guissard:** data curation, conceptualization, investigation. **Carole Sanchez:** conceptualization, investigation, data curation. **Virginie Paulus:** data curation, software, methodology, conceptualization, investigation. **Monique Henket:** conceptualization, investigation, methodology, software, data curation. **Genevieve Philippe:** methodology, conceptualization. **Casper W. H. Beijnink:** software, data curation, methodology. **Pierre‐Olivier Bridevaux:** conceptualization, methodology, software, writing – review and editing, data curation, supervision, resources, validation, formal analysis. **Florence Schleich:** conceptualization, methodology, validation, data curation, supervision, resources. **Renaud Louis:** conceptualization, validation, visualization, writing – review and editing, methodology, software, formal analysis, project administration, resources, supervision, data curation.

## Funding

The authors have nothing to report.

## Conflicts of Interest

The authors declare no conflicts of interest.

## Supporting information


Supporting Information S1

## Data Availability

The data that support the findings of this study are available from the corresponding author upon reasonable request.
