# Supplementary material for: Clinical Remission in Severe T2‐High Asthma in Real Life After Anti‐IgE, Anti‐IL‐5 and Anti‐IL5R: A Potential Role for CRP as a Biomarker
Source: Clin Transl Allergy. 2026 Apr 15;16(4):e70164. doi: 10.1002/clt2.70164 (PMC13084146; doi:10.1002/clt2.70164)
Supplement: Supplementary file 1 — Supporting Information S1 [file CLT2-16-e70164-s001.docx]

**Supplementary Figure 1 :** Reimbursement Criteria for Biologic Therapies Defined by the INAMI (National Institute for Health and Disability Insurance – Belgium)


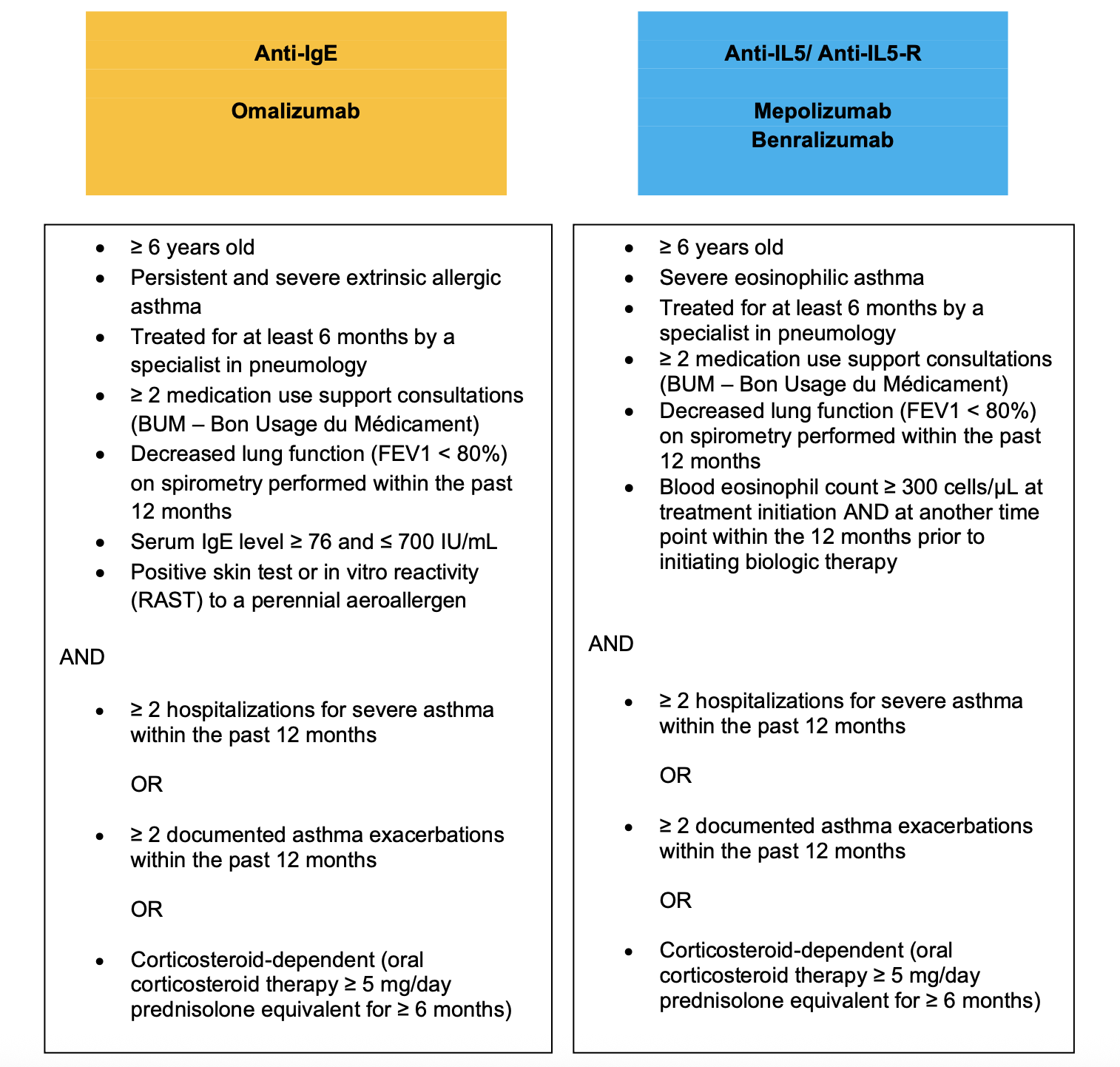


**Supplementary Table 1 :** Baseline characteristics of severe asthma patients treated with anti-IgE vs anti-IL-5 and anti-IL-5 R

|  | Group  anti-IgE  Omalizumab  (N = 97) | Group anti-IL5 / anti-IL5R  Mepolizumab  Benralizumab  (N = 109) | P value |
| --- | --- | --- | --- |
| Demographics |  |  |  |
| Age (y), mean +/- SD | 50 ± 16 | 54 ± 15 | 0.025 |
| Gender (male,n, %) | 37 (38) | 44 (40) | 0.744 |
| BMI (kg/m^2^), mean +/- SD | 28 ± 5 | 27 ± 5 | 0.282 |
| Atopy (n,%) | 65 (77) | 33 (36) | <0.001 |
| Smoking status (%) |  |  |  |
| Never (n,%) | 48 (49) | 61 (56) | 0.019 |
| Ex (n,%) | 33 (34) | 43 (39) |  |
| Current (n,%) | 16 (17) | 5 (5) |  |
| Age at onset (y), mean +/- SD | 23 ± 19 | 41 ± 19 | <0.001 |
| Duration of asthma (y), mean +/- SD | 25 ± 13 | 13± 18 | 0.006 |
| Level of asthma control |  |  |  |
| OCS burst in past year (n), mean +/- SD) | 2.0 ± 2.0 | 3.3 ± 2.0 | <0.001 |
| Hospitalisation (n), mean +/- SD) | 0.3 ± 0.7 | 0.3 ± 0.7 | 0.932 |
| ACT score, mean +/- SD | 12 ± 5 | 12 ± 5 | 0.374 |
| ACQ6 score, mean +/- SD | 2.8 ± 1.0 | 2.8 ± 1.4 | 0.978 |
| Pulmonary function |  |  |  |
| FEV1 (%predicted Pre BD) | 66 ± 18 | 69 ± 19 | 0.191 |
| FEV1 (%predicted Post BD) | 71 ± 19 | 75 ± 20 | 0.248 |
| FVC (%predicted (Pre BD) | 79 ± 16 | 82 ± 18 | 0.182 |
| FVC (%predicted Post BD) | 81 ± 17 | 85 ± 18 | 0.174 |
| FEV1/FVC Pre BD (%) | 69 ± 12 | 69 ± 12 | 0.946 |
| BDR (% baseline) | 9 ± 12 | 9 ± 12 | 0.921 |
| Inflammatory biomarkers |  |  |  |
| Geometric mean of FeNO (ppb), mean +/- SD log^e^ scale | 27.03 ± 1.10 | 34.56 ± 0.85 | 0.127 |
| Geometric mean sputum neutrophils (%),mean +/- SD on log^e^ scale | 59 ± 26 | 54 ± 29 | 0.351 |
| Geometric mean sputum eosinophils (%), mean +/- SD on log^e^ scale | 12 ± 18 | 25 ± 28 | 0.003 |
| Geometric mean blood neutrophils count (1/µL), mean +/- SD on log^e^ scale | 4.76 ± 0.39 | 4.64 ± 0.35 | 0.586 |
| Geometric mean blood eosinophils count (1/µL) , mean +/- SD on log^e^ scale | 206.31 ± 1.39 | 462.86 ± 0.76 | <0.001 |
| Fibrinogen (g/L), mean +/- SD | 3.8 ± 1.2 | 3.6 ± 1.0 | 0.380 |
| Geometric mean CRP (mg/L), mean +/- SD on log^e^ scale | 2.91 ± 1.01 | 2.69 ± 1.38 | 0.335 |
| Geometric mean total IgE (kU/L), mean +/- SD on log^e^ scale | 233.02 ± 0.98 | 118.77 ± 1.49 | 0.001 |
| Treatment |  |  |  |
| EQ_Beclometasone (mcg/j), mean +/- SD | 2315 ± 989 | 2230 ± 945 | 0.553 |
| ICS, (n,%) | 95 (98) | 109 (100) | 0.132 |
| LABA, (n, %) | 93 (96) | 106 (97) | 0.588 |
| LAMA, (n, %) | 16 (17) | 21 (19) | 0.605 |
| SAMA, (n, %) | 46 (47) | 40 (37) | 0.119 |
| SABA, (n, %) | 73 (75) | 73 (67) | 0.191 |
| THEO, (n, %) | 4 (4) | 4 (4) | 0.866 |
| LTRA, (n, %) | 56 (58) | 46 (42) | 0.026 |
| HRA, (n, %) | 26 (27%) | 19 (17%) | 0.104 |
| OCS, (n, %) | 22 (23%) | 30 (28%) | 0.425 |

Results are expressed as mean± SD, geometric mean± SD for inflammatory biomarkers, or median (range) for ICS dosage

*EQ = equivalent, FeNO = fractional exhaled nitric oxide ; FEV1 = forced expiratory volume, HRA = histamine releasing activity, ICS = inhaled corticosteroids, LABA = long acting beta agonist, LAMA = long acting muscarinic antagonist, LTRA = leukotriene receptor antagonist, OCS = oral corticosteroids, SABA = short acting beta agonist, SAMA = short acting muscarinic antagonist, THEO = theophylline*
